# Supplementary material for: Arcuate nucleus and lateral hypothalamic CART neurons in the mouse brain exert opposing effects on energy expenditure
Source: eLife. 2018 Aug 21;7:e36494. doi: 10.7554/eLife.36494 (PMC6103747; doi:10.7554/eLife.36494)
Supplement: Supplementary file 1. [file elife-36494-supp1.docx]

**Supplementary File 1. Sequences of oligonucleotide primers used in qPCR**

| Gene symbol | 5’ Oligonucleotide | 3’ Oligonucleotide |
| --- | --- | --- |
| *Cartpt* | AAACGCATTCCGATCTACGA | TCACAAGCACTTCAAGAGGA |
| *Gad1* | CACCGAGCTGATGGCATCT | AGATCTTCAGGCCCAGTTTT |
| *Gad2* | GATGTCAACTACGCGTTTCT | AAGCTCATTGGGGTAATGGA |
| *Gal* | TGGCTCCTGTTGGTTGTGA | TTTCCTCCACCTCCAGTTG |
| *Hcrt* | GTCTCTACGAACTGTTGCACG | GCTAAAGCGGTGGTAGTTAC |
| *Lepr* | AAGACACTGGCTTCAGTAGT | TTCTTGAAGGGGTTCTTAGG |
| *Nts* | CTTCCAGCTCCAGAAAATCT | AGGGCCTTCTGGGTTTATTT |
| *Pmch* | CCCTTCTCTGGAACAATACA | TCAGAGCGAGGTAAGGTTTT |
| *Slc32a1* | GGAACGTGACAAATGCCATT | CCTGTACGAGGAGAACGAA |
| *Actb* | AGCACCCTGTGCTGCTCA | GTACGACCAGAGGCATACA |
